# Gut Microbiota Alterations in Trace Amine-Associated Receptor 9 (TAAR9) Knockout Rats

**DOI:** 10.3390/biom12121823

**Published:** 2022-12-06

**Authors:** Ilya S. Zhukov, Anastasia N. Vaganova, Ramilya Z. Murtazina, Lyubov S. Alferova, Elena I. Ermolenko, Raul R. Gainetdinov

**Affiliations:** 1 Institute of Translational Biomedicine, Saint Petersburg State University, Universitetskaya nab. 7/9, 199034 Saint Petersburg, Russia; 2 Institute of Experimental Medicine, Acad. Pavlov str. 12, 197376 St. Petersburg, Russia; 3 St. Petersburg University Hospital, Saint Petersburg State University, Universitetskaya nab. 7/9, 199034 Saint Petersburg, Russia

**Keywords:** trace amine-associated receptor, TAAR, TAAR9, gut microbiota, dopamine, *Saccharimonadaceae*, transcriptomic data

## Abstract

Trace amine-associated receptors (TAAR1-TAAR9) are a family of G-protein-coupled monoaminergic receptors which might have great pharmacological potential. It has now been well established that TAAR1 plays an important role in the central nervous system. Interestingly, deletion of TAAR9 in rats leads to alterations in the periphery. Previously, we found that knockout of TAAR9 in rats (TAAR9-KO rats) decreased low-density lipoprotein cholesterol levels in the blood. TAAR9 was also identified in intestinal tissues, and it is known that it responds to polyamines. To elucidate the role of TAAR9 in the intestinal epithelium, we analyzed TAAR9-co-expressed gene clusters in public data for cecum samples. As identified by gene ontology enrichment analysis, in the intestine, TAAR9 is co-expressed with genes involved in intestinal mucosa homeostasis and function, including cell organization, differentiation, and death. Additionally, TAAR9 was co-expressed with genes implicated in dopamine signaling, which may suggest a role for this receptor in the regulation of peripheral dopaminergic transmission. To further investigate how TAAR9 might be involved in colonic mucosal homeostasis, we analyzed the fecal microbiome composition in TAAR9-KO rats and their wild-type littermates. We identified a significant difference in the number of observed taxa between the microbiome of TAAR9-KO and wild-type rats. In TAAR9-KO rats, the gut microbial community became more variable compared with the wild-type rats. Furthermore, it was found that the family Saccharimonadaceae, which is one of the top 10 most abundant families in TAAR9-KO rat feces, is almost completely absent in wild-type animal fecal samples. Taken together, these data indicate a role of TAAR9 in intestinal function.

## 1. Introduction

Until recently, the majority of the family of receptors termed trace amine-associated receptors (TAARs) were considered to be almost exclusively expressed in the olfactory epithelium (except TAAR1) [1] and to participate in sensing socially relevant innate odors [2]. G-protein-coupled TAAR receptors were named for their ability to recognize low-abundance biogenic amine neurotransmitters termed trace amines [3]. Currently, it is evident that TAARs can be activated both by trace amines and other endogenous and exogenous amine molecules [1,4]. While TAAR1 expression and function in the brain have been vigorously established [1,4], the role of other receptors in the regulatory processes beyond olfaction, especially outside the brain, remains poorly understood.

Trace amines and their receptors may play significant roles in the function of the gut microbiota of mammals [5]. It is well established that beta-Phenylethylamine (PEA), p-Tyramine (TYR), tryptamine (TRP), and other endogenous trace amines are commonly found in anaerobic fermented products [6,7,8,9]. Classical examples of such products are aged cheeses, fermented meats, red wine, soy products, and chocolate [10]. The famous “cheese effect” in patients treated with monoamine oxidase inhibitors is explained by pronounced accumulation of TYR, which is sufficient to indirectly elevate blood norepinephrine concentrations to the point of inducing a hypertensive crisis, severe migraine, and even death [11]. TAAR9 mRNA and other TAARs were identified in the mucosal layer of the gastrointestinal tract. TAAR9 is expressed mainly in the duodenum; its expression in the upper intestine, lower intestine, and proximal colon is lower [12,13,14]. Despite the fact that TAAR9 expression in the gastrointestinal tract was identified over a decade ago, its function in this organ system still remains unidentified.

TAAR9 is also known to be expressed at a low level in immune cells, including NK-cells, T-cells, B-cells, and monocytes [15]. TAAR1 and TAAR9 elimination in knockout rodents is associated with minor deregulation of lipid and protein metabolism [16], which becomes more pronounced under a high-carbohydrate diet [17]. Interestingly, total cholesterol and low-density lipoproteins are decreased in TAAR9-KO animals. The cause of these differences is unknown but may be related to a decreased endogenous cholesterol absorption, biosynthesis, transport, or any other alterations in metabolism [18]. This receptor can sense amines such as N, N-dimethylcyclohexylamine, N-methylpiperidine, triethylamine, and polyamines, such as cadaverine, putrescine, spermidine, and spermine [19,20,21].

Putrescine is the most abundant polyamine in the human colon followed by spermine, spermidine, and cadaverine [22]. These polyamines are involved in a plethora of biological functions, including gene expression regulation, stress resistance, cell proliferation, and differentiation. In the gastrointestinal tract, these compounds take part in mucosa homeostasis, intercellular junctions, cell division, cell migration after wounding, and apoptosis [22,23,24,25,26,27]. Polyamines also modulate systemic and mucosal adaptive immunity [24,28,29,30]. Decreased endogenous polyamine synthesis might be involved in intestinal hypoplasia [22] and the development or exacerbation of inflammatory bowel disease [31]. Meanwhile, high concentrations of polyamines are linked to intestinal epithelium malignization [24,32,33]. However, both putrescine and cadaverine may damage the colon epithelium and become cytotoxic at concentrations found in biogenic amine-rich foods [34,35]. Additionally, endogenous polyamines are synthesized in epithelial cells [36]. Cationic properties of polyamines determine their interactions with intracellular and extracellular acidic residues, including nucleic acids, phospholipids, acidic proteins, and carboxyl- or sulfate-containing polysaccharides [36]. Thus, polyamines have multifactorial effects on biomolecules. For example, it was shown that polyamines stimulate transcription of the growth-promoting genes, but they inhibit growth-inhibiting gene expression at the post-transcriptional level [26].

The role of TAAR9 in polyamine sensing and polyamine-mediated interaction between enterocytes and the gut microbiota have not yet been investigated. To reveal the role of TAAR9 in the colonic mucosa, we analyzed its expression in public transcriptomic data. Additionally, to determine TAAR9′s contribution to maintaining the gut microbiota composition, the TAAR9-KO fecal bacterial community was studied using high-throughput 16S-rDNA sequencing and compared with the fecal microbiome of wild-type littermates.

## 2. Materials and Methods

### 2.1. Public Transcriptome Data Analysis

Transcriptomic data for the TAAR9 expression were searched in the Expression Atlas database [37] for the term “TAAR9”. As no relevant data for the rat were identified, the dataset “RNA-seq of mouse intestinal mucosa to investigate infection by the parasitic nematode Trichuris muris” [38], dataset ID E-ERAD-181, was selected for further analysis. Transcriptomic data for the cecum samples of the control non-infected group of C57BL/6 mice were downloaded.

Data were normalized by the CPM (count per million) method in the edgeR [39] R package. The genes co-expressed with TAAR9 were identified by Pearson’s correlation. The top 100 co-expressed genes were selected for gene ontology (GO) enrichment analysis. GO enrichment analysis (identification of GO terms that are significantly enriched by the genes of the selected set) and visualization of results were performed using the ShinyGo 0.76 [40] web tool (available at http://bioinformatics.sdstate.edu/go/, accessed on 30 August 2022). The “GO Biological Process” database was used for the enrichment analysis.

This section may be divided by subheadings. It should provide a concise and precise description of the experimental results, their interpretation, as well as the experimental conclusions that can be drawn.

### 2.2. Animals and Sample Collection

TAAR9-KO rats were generated using CRISPR/Cas9-mediated mutagenesis [18]. A line of TAAR9-KO rats with single nucleotide deletion (TAAR9-KO^delC^) was studied. Breeding and genotyping of knockout and wild-type rats were performed as described previously [18]. Rats were maintained on a 12:12 h light/dark cycle with a light on at 08:00 h and were allowed access to food and water ad libitum. Three-month-old Sprague Dawley TAAR9-KO rats (TAAR9-KO^delC^ strain, *n* = 8) and wild-type (WT, *n* = 8) littermates were used in the study. Fecal samples were collected in 1.5-mL Eppendorf tubes early in the morning and were immediately frozen at −80 °C.

All procedures were performed under the guidelines established by the European Community Council (Directive 2010/63/EU of 22 September 2010), and animal protocols were approved by the Ethics Committee of St. Petersburg State University, St. Petersburg, Russia.

### 2.3. Gut Bacterial DNA Extraction and Sequencing

Fecal samples (100 mg) were used for DNA extraction with the NucleoSpin Soil MACHEREY-NAGEL (Germania) kit according to the manufacturer’s protocol. The V3-V4 region of the 16S rRNA was amplified with the sequencing primers (positions based on *E. coli* SSU rRNA numbering): F9, F338 (59-ACICCTACGGGIGGCAGCAG-39; 338 to 357), and R805 (59-GACTACCCGGGTATCTAATCC-39; 805 to 785). These primers target the V3-V4 hypervariable region of bacterial 16S rRNA genes. PCR was then performed under the following conditions: denaturation (95 °C, 3 min); amplification (25 cycles), annealing (55 °C, 30 s), elongation (72 °C, 30 s), and final elongation (72 °C, 5 min). Sequencing was performed on the platform of Illumina HiSeq 2000.

### 2.4. Sequencing Data Processing

The raw data of 16S rRNA paired-end reads were cut with forward and reverse primers using the QuasR R package [41], and the trimmed sequences were processed using the DADA2 (Callahan et al., 2016) pipeline to generate an amplicon sequence variant (ASV) table. The paired-end fastq files were merged, and redundancy was removed. Each ASV was annotated with the SILVA high-quality ribosomal RNA database (realize 138) [42].

### 2.5. Bioinformatics and Statistical Analysis of 16S rRNA Sequencing Data

The dataset was analyzed using the R package MicrobiotaProcess [43]. Diversity indices, including observed species, abundance-based coverage estimators (ACE) index, Chao1 index, Shannon diversity index, Simpson index, and Pielou’s evenness, were calculated for each sample and compared between WT and TAAR9-KO animals by the Wilcoxon test. Diagrams were produced with the R packages MicrobiotaProcess [44] and ggplot2 [45].

β-diversity, which estimates the difference in community structure between samples, was measured using the Bray–Curtis distance based on an evenly rarefied abundance table. The distance between the samples was plotted with a box plot. Statistical differences in the measured β-diversity metrics across groups were determined by the permutational multivariate analysis of variance (PERMANOVA) with distance matrices using the adonis command in the R package vegan [45]. Β-diversity was visualized via principal coordinate analysis (PCoA). The difference between the samples was evaluated by hierarchical cluster analysis.

Taxa with differential abundances in the groups were identified by the Wilcoxon Kruskal–Wallis test in the R package MicrobiotaProcess, which is an algorithm for biomarker discovery based on the tidy-like framework.

Pearson’s correlations were calculated, and the correlation matrix was visualized by the corrplot [46] R package.

## 3. Results

### 3.1. Analysis of TAAR9 Expression and TAAR9 Co-Expressed Gene Cluster in Colon Tissue

**Figure 1:**
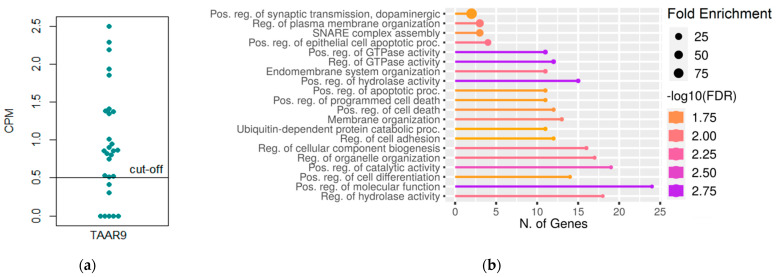
TAAR9 expression in mouse cecum. (**a**) TAAR9 mRNA expression in non-treated C57BL/6 mice (dataset E-ERAD-181); (**b**) gene ontology enrichment analysis of top 100 TAAR9 co-expressed genes. CPM—count per million, FDR—false discovery rate.

The dataset E-ERAD-181 located in the public database Expression Atlas includes transcriptomic data for 28 cecum samples from wild-type, non-infected mice. To interpret numerical values, we used Expression Atlas terms [37], i.e., “expression level is below cut-off” if the expression value <0.5 CPM and “low expression level” if CPM is between 0.5 and 10. Applying these terms for description, 21 cecum samples (75%) were considered to have TAAR9 expression at a low level (Figure 1a).

As RNAseq provides detailed information about the expression landscape of multiple genes in the biological samples, we identified genes co-expressed with TAAR9 in the cecum. Considering that co-expressed genes are involved in direct or indirect interactions, share common functions [47,48], and frequently are regulated by the same mechanisms [49], we selected the top 100 genes co-expressed with TAAR9 (Supplementary Figure S1) in cecum samples and performed the GO enrichment analysis to identify their function. The identified TAAR9 co-expressed genes were involved in membrane organization, regulation of GTPase activity, hydrolase activity, cell death, adhesion, and differentiation (Figure 1b). Specifically, the involvement of TAAR9 co-expressed genes in the SNARE complex assembly and dopaminergic signaling was demonstrated.

### 3.2. Fecal Microbiota α-Diversity in TAAR9-Ko Rats and Wild-Type Littermates

Cumulatively, 40,833 high-quality sequences from 16 samples were received, with an average of 2552 sequences per sample. Overall, 708 taxa were identified in the 16 samples.

**Figure 2:**
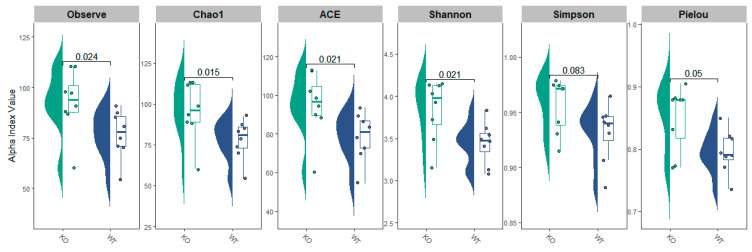
α-diversity among wild-type (WT) and TAAR9-knock out (KO) rats. α-diversity, measured by observed species, Chao1, abundance-based coverage estimator (ACE), Shannon, Simpson, and Pielou results are plotted for examined samples. Box plots and violin plots depict microbiome diversity and abundance differences according to each test. The horizontal line inside the box represents the median. Individual sample values are represented by dots.

The α-diversity was characterized by several metrics. The microbial communities’ richness was characterized by the observed species index, Chao1, and ACE indexes, and Shannon and Simpson’s indexes were then implemented to estimate microbiome evenness and homogeneity. Pielou’s evenness, which is Shannon diversity normalized by the observed richness, was calculated. A higher microbial richness was identified in TAAR9-KO mice compared with wild-type animals (*p* < 0.05). Conversely, the results for the microbial communities’ evenness seemed to be controversial. Shannon index and Pielou index were higher in the TAAR9-KO rats (*p* < 0.05), which suggests a higher diversity of the fecal microbial community in this group, but there were no significant differences in the Simpson index values between the two groups (*p* = 0.083; Figure 2).

### 3.3. Difference in β-Diversity of Fecal Microbiota Species between TAAR9-Ko Rats and Wild-Type Littermates

**Figure 3:**
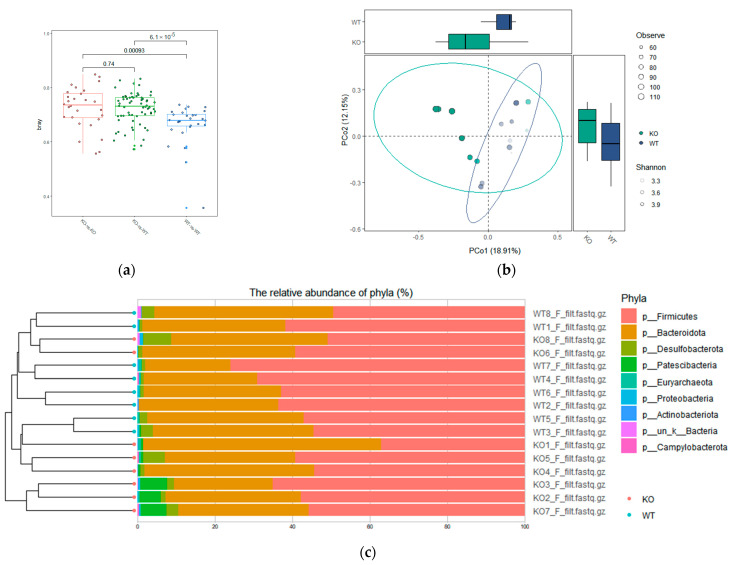
β-diversity among wild-type (WT) and TAAR9-knockout (KO) rats. (**a**) The comparison of Bray–Curtis distance among the groups; (**b**) PCoA of the microbiota composition showing significant difference (Bray–Curtis distances) between TAAR9-KO and wild-type rats (*p* = 0.0274); (**c**) the hierarchical cluster result of TAAR9-KO and wild-type samples.

The β-diversity of the gut microbiota was evaluated by PCoA based on the Bray–Curtis distance matrix at the level of phylum. Bray–Curtis distances within the TAAR9-KO group, within the wild-type group, and between the TAAR9-KO and wild-type control were plotted to compare fecal microbiota dissimilarity in the different genetic backgrounds (Figure 3a). Interestingly, the dissimilarity between the TAAR9-KO samples was comparable to the dissimilarity between TAAR9-KO and wild-type samples (*p* = 0.74). The dissimilarity between the samples from the wild-type animals was significantly (*p* < 0.001) lower than between TAAR9-KO samples or between TAAR9-KO and wild-type samples.

PCoA of the microbiota composition data demonstrated that the microbiota of TAAR9-KO rats was distinct from that of wild-type animals, although there was overlap (*p* = 0.0274, Figure 3b). The hierarchical cluster result of the samples (Figure 3c) confirmed some overlap between the study groups. Most TAAR9-KO samples from the cluster separated from the cluster of wild-type samples, but two TAAR9-KO samples were more similar to wild-type samples, and two wild-type samples were clustered with TAAR9-KO samples.

### 3.4. Differential Abundances of Bacterial Taxa

**Figure 4:**
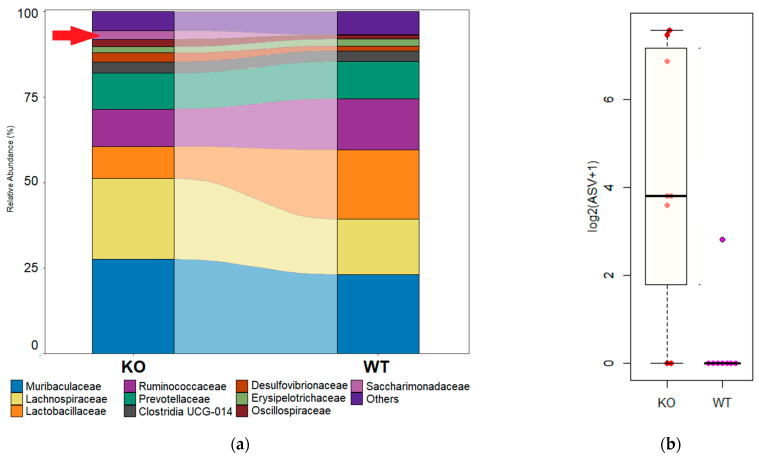
Microbiome analysis revealed higher *Saccharimonadaceae* prevalence in the fecal microbiota of TAAR9-KO compared with wild-type littermates. (**a**) The averaged relative abundance at the family level in TAAR9-KO and wild-type rats (top 10 most abundant families are represented); (**b**) the abundance of *Saccharimonadaceae* in fecal microbiome in TAAR9-KO and wild-type rats.

Despite the pronounced differences in fecal microbial communities between TAAR9-KO and wild-type animals, the differential abundance analysis at the levels of the genus, order, or phylum did not demonstrate significant differences between the study groups. The relative abundance was significantly modified by the TAAR9-KO only for one family, i.e., the family Saccharimonadaceae (*p* < 0.01, Figure 4a, Supplementary Figure S1). This sole difference between the study groups did not reach statistical significance after the FDR adjustment. Despite the demise of statistical significance after the adjustment, the family Saccharimonadaceae is one of the top 10 families in the TAAR9-KO rats, but it is almost completely lost in wild-type animals (it is absent in all but one sample, Figure 4b).

### 3.5. Asv—Amplicon Sequence Variant; KO—TAAR9-KO Group; Wt—Wild-Type Control Group

Because *Saccharimonas spp.* are considered to be epibiotic bacteria, we performed a correlation analysis between the *Saccharimonas* genus and other genera identified in TAAR9-KO rat feces. The richness of the *Saccharimonas* genus was correlated with the abundance of various species (Figure S2). The most pronounced correlation levels were identified between *Saccharimonas* and *Ligilactobacillus* (r = 0.957, *p* = 2.846213 × 10^−45^), Tyzzerella (r = 0.888, *p* = 3.511427 × 10^−34^), and an unknown genus of the family Lactobacillaceae (r = 0.953, *p* = 2.555605 × 10^−40^).

## 4. Discussion

TAAR9 expression in the colon was identified previously [12,13,14]. However, the biological significance of this receptor in the colon remains uncertain. TAAR9 expression in colon tissue, particularly in the mouse cecum, was confirmed by the analysis of public transcriptomic data represented in the Expression Atlas [37] database. The analysis of TAAR9 co-expressed genes showed the association between TAAR9 expression in colon tissue and producing the proteins involved in fundamental processes of tissue homeostasis, including adhesion, cell differentiation, and cell death.

Additionally, a relationship between TAAR9 and proteins involved in membrane organization, including the SNARE complex, was identified. SNARE complex proteins contribute to brush border protein trafficking in enterocytes [50] and the secretory function of L-cells of the colon mucosa [51], and SNARE proteins also contribute to the lipid transfer through the enterocytes in the small intestine [52]. Moreover, SNARE proteins contribute to the interaction with extracellular matrix and cell-cell contacts [53]. Interestingly, it was previously demonstrated that TAAR9 expression may be related to decreased endogenous cholesterol absorption, biosynthesis, transport, or any other alterations in metabolism [17].

Of particular interest is the TAAR9 co-expression of genes involved in dopaminergic signaling. The role of another trace amine-associated receptor, TAAR1, in the modulation of dopamine signaling is well-studied both in the nervous system and outside the neural tissues [54]. After the dimerization of TAAR1 with the dopamine receptor D2R, dopamine signaling activity shifts from the β-arrestin-2 signaling pathway to Gi activation [1,55,56,57,58]. The study of TAAR5-KO animals and TAAR5 agonist α-NETA also demonstrated effects of TAAR5 absence on dopaminergic transmission functioning, at least in the central nervous system [59,60,61,62,63]. In contrast, the role of TAAR9 in dopaminergic transmission was not described. The identified association suggests possible participation of TAAR9 in gut dopamine signaling, although it cannot explain the mechanism of this participation. It is well established that dopamine in the digestive tract is essential for secretion and absorption, mucosal homeostasis, regeneration, and protection [64,65].

TAAR9 can be activated by various amines, such as N, N-dimethylcyclohexylamine, N-methylpiperidine, and triethylamine, and polyamines, especially cadaverine, putrescine, spermidine, and spermine [19,20,21]. Dietary polyamines are absorbed in the small intestine, and the gut microbiota, primarily, Bacteroides spp., Fusobacterium spp., and Clostridia [66], is the major source of polyamines in the lower part of the intestine [22,24,27,67,68]. Polyamine synthesis pathways seem to be generated in the gut microbial community, where enzymes produced by different species of bacteria collectively enable the synthesis of polyamines from precursors [30,69]. The production of polyamines by the gut microbiota likewise depends on dietary fermentable carbohydrates [27], fibers [68], and proteins [22]. Luminal bacterium-derived polyamines facilitate colonic epithelial proliferation. The anti-inflammatory role of bacterium-derived polyamines in the intestinal immune system was also demonstrated [31]. Thus, polyamines have multifactorial effects on biomolecules and the intestinal microbiota.

Polyamines, including putrescine, spermidine, spermine, and cadaverine, in the lower parts of the intestine are considered to be synthesized by the gut microbiota [70]. These compounds are involved in the interactions between bacteria and impact intestinal health, including the maintenance and recovery of colon barrier function [27]. Polyamines regulate biological processes in cells in different ways, including direct interaction with DNA, RNA, nucleosomes, inward rectifier K+ (Kir) channel pores or NMDA receptor, β- and γ-phosphates of ATP, protein kinases, and eukaryotic translation initiation factor 5A (eIF5A) [71,72]. Several polyamine effects, such as the modulation of immune system activity and inflammation [73] or protective action from the bacterial toxins in colon mucosa [74], may be significant for host–microbial interactions in the colon. Many of the predominant species of the indigenous human gut microbiota are capable of taking up and/or exporting polyamines [72]. On the other hand, polyamines are also crucial for the virulence phenotype of many bacterial pathogens [74]. The role of GPCR-mediated mechanisms, including TAAR9-dependent signaling regulating polyamine biological activity (such as polyamine sensing by enterocytes), remains unexplored and requires detailed investigation in future studies.

The observed changes in the gut microbiota composition may reflect TAAR9 participation in maintaining the stability of the gut microbial community. Individual variations were significantly more pronounced in the TAAR9-KO group compared with the wild-type littermates. The key elements of biodiversity include the richness of local and global species; genetic diversity of populations and species; the spatial extent and the state of natural habitats; and the functioning of ecosystems that are essential for mankind to survive. Increasing ecosystem diversity promotes stability through various mechanisms, such as functional redundancy, broader utilization of available resources, weak among-species interactions and alternative energy channels [75].

The differential abundance analysis did not reveal any taxa that may be considered as a biomarker of TAAR9-KO rat fecal microbiota with any statistical significance. However, we revealed that family Saccharimonadacea and the genus *Saccharimonas* were represented only in TAAR9-KO samples and were almost not detected in the samples from wild-type rats.

The family Saccharimonadaceae of the phylum “Candidatus Saccharibacteria” are considered obligate epibionts on the surface of other bacteria [76] including Bacteroidetes, Actinobacteria, and Proteobacteria [77]. Saccharibacteria is characterized by small cell size (200–300 nm in diameter), small genome size, and limited metabolic, especially biosynthetic, capacities [76,77,78,79]. These bacteria produce a variety of catabolic enzymes that degrade complex biomolecules and numerous copies of different ABC transporters [77,80]. Additionally, the genes predicted to confer resistance to bacterially produced antibiotics were identified in the genomes of Saccharimonadaceae [80].

Currently, an unambiguous association between Saccharimonadaceae abundance in the colon microbiota and health status was not identified. Consumption of dietary supplements enriched with natural antioxidants or polysaccharides [81,82,83,84], probiotic strain Lactiplantibacillus plantarum-12 [85], or fecal microbiota transplantation in dysbiotic animals [85] elevates the abundance of Saccharimonadaceae. Conversely, the abundance of Saccharimonadaceae decreases in the obese rat [82,86] and was negatively correlated with non-alcoholic fatty liver disease scores in model conditions [87]. In humans, a high intake of dietary saturated fatty acids led to the depletion of Saccharimonadaceae in the gut microbiota [88]. Chronic concurrent exposure to inorganic arsenic and fluoride was also associated with the loss of Saccharimonadaceae from the gut microbiome in rats [89]. However, Saccharimonadaceae richness decreased after the dietary consumption of prebiotic isomaltulose [90], some synbiotic compositions [91], and natural compounds with restorative strengthening effects [92,93,94], and it may increase in response to treatment with carcinogenic compounds [95] or after long-term proton pump inhibitor exposure, which is damaging for the colonic mucosa [96]. The Saccharimonadaceae family is overrepresented in patients with lupus erythematosus [97], and carriers of HLA alleles are associated with a higher risk of autoimmune diseases [98].

Identification of genera that were co-abundant with *Saccharimonas sp.* in the TAAR9-KO group could not resolve the controversy regarding the significance of the presence of candidatus Saccharimonadaceae in the colon. Both genera Ligilactobacillus and Tyzzerella belong to the phylum Firmicutes, although the impact of these taxa on the host health is different. Several stains of Ligilactobacillus sp belonging to the Lactobacillaceae family demonstrate probiotic properties [99,100,101,102]. In contrast, representatives of the genus Tyzzerella might be pathogenic [103,104] or associated with a high inflammatory background [105,106].

Polyamines have multiple effects on the intestine epithelium, including host–microorganism interactions with commensal gut bacteria [27]. It is possible that TAAR9, as a receptor sensitive to polyamines, is involved in these interspecies communications. The current study identified some involvement of TAAR9 in intestinal epithelium homeostasis and interaction with the gut microbiota, although it is not without limitations. GO enrichment analysis in the set of TAAR9 co-expressed genes is based on statistical simulations, so it does not provide information on the actual role of TAAR9 in the identified biological processes. Moreover, the low depth of 16S rRNA gene sequencing provides the data for the most abundant bacteria in the samples. As a consequence, information for rare microbial genera is lost or insufficient. Direct assessment of the changes in polyamine production in the gut microbial community in response to the disruption of TAAR9 in rats also was not performed. The differences in microbiome structure between TAAR9-KO and wild-type littermates also may reflect some changes in the metabolic or immune status of TAAR9-KO animals instead of the dysfunction of TAAR9-dependent mechanisms of gut microbial community control. Further examination of TAAR9 function and TAAR9-KO animals could help to determine the biological base of differences identified in the present study.

## 5. Conclusions

In this study, we identified genes co-expressed with TAAR9 in colon tissues, and several genes were identified that are involved in biological processes associated with colon epithelium functioning and homeostasis, including dopaminergic transmission. This association could imply a role of TAAR9 in monoaminergic signaling as was described for other trace amine-associated receptors such as TAAR1, TAAR2, or TAAR5 [59,107,108,109]. Additionally, TAAR9 seems to be significant to microbiota homeostasis. In TAAR9-KO rats, the microbiome structure became more variable than in wild-type littermates. Even though the alpha diversity was higher in the TAAR9-KO fecal microbial community, no specific taxa were significantly overrepresented in this group. However, it should be noted that the family Saccharimonadaceae, which is absent in the wild-type animal microbiome, became one of the top 10 families in the TAAR9-KO group. As these bacteria are symbionts of other bacterial taxa, the increase in Saccharimonadaceae abundance in TAAR9-KO rats may reflect some perturbations in the colon microbial community, which was not investigated in this paper. Further studies are necessary to reveal the contribution of TAAR9 to peripheral dopamine signaling and the link between TAAR9 and the intestinal microbial community.

## Data Availability

All of the data is presented in the article and Supplementary Materials. No additional data is reported.

## Supplementary Materials

The following supporting information can be downloaded at: https://www.mdpi.com/article/10.3390/biom12121823/s1. Figure S1: Cladogram for all subjects and 17 taxa showing insignificant changes between the wild-type (WT) and TAAR9-KO (KO) rats. Figure S2: Pearson’s correlation by genus abundance in the fecal microbiome of TAAR9-KO rats. Significant values (*p* < 0.05) are shown by cyrcles.
