# Supplementary material for: Gut Microbiota Alterations in Trace Amine-Associated Receptor 9 (TAAR9) Knockout Rats
Source: Biomolecules. 2022 Dec 6;12(12):1823. doi: 10.3390/biom12121823 (PMC9775382; doi:10.3390/biom12121823)
Supplement: Supplementary file 1 [file biomolecules-12-01823-s001.zip › biomolecules-2025084-supplementary.pdf]

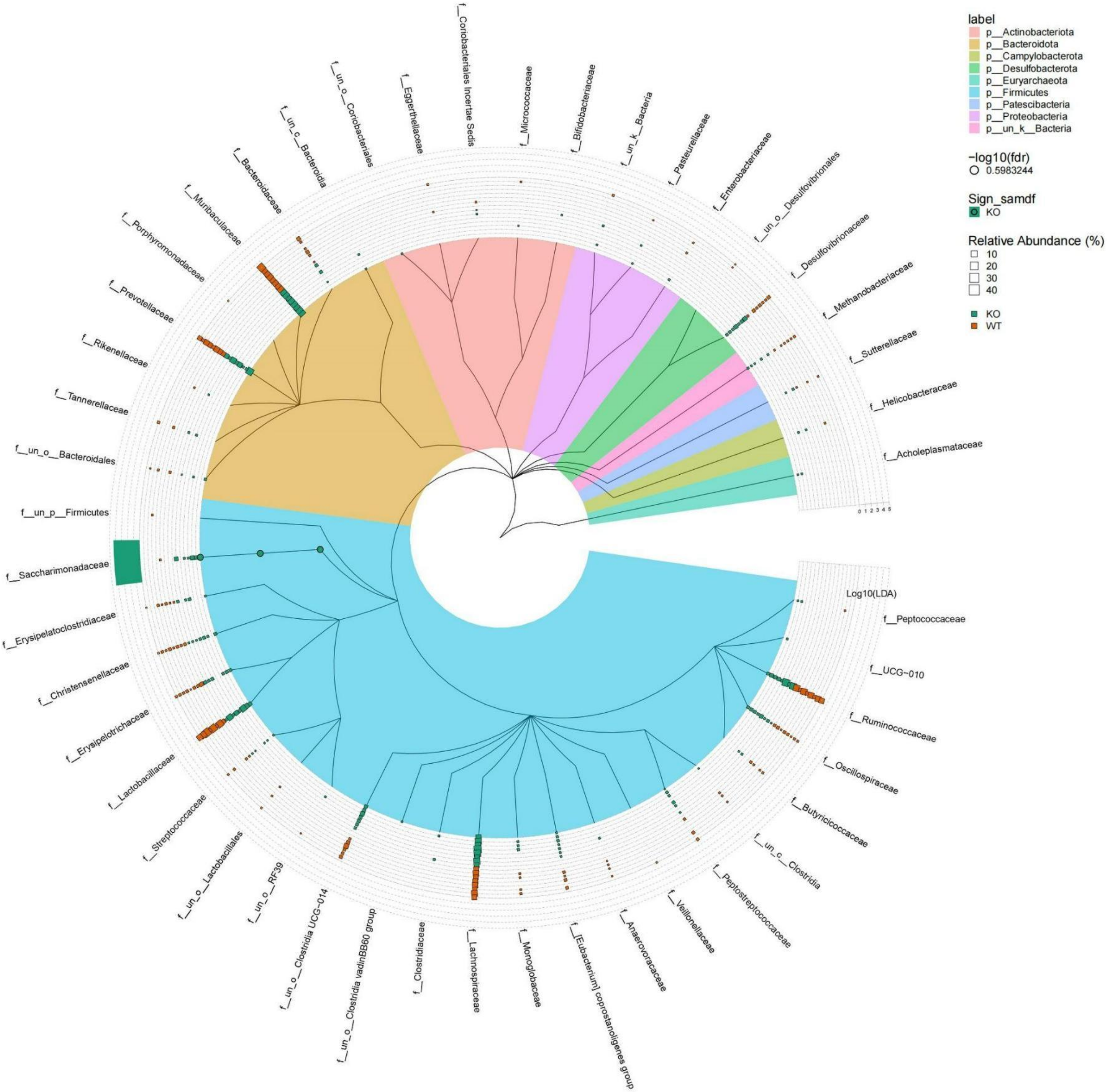

Supplementary Figure S1

Cladogram for all subjects and 17 taxa showing insignificant changes between the wild-type (WT) and TAAR9-KO (KO) rats

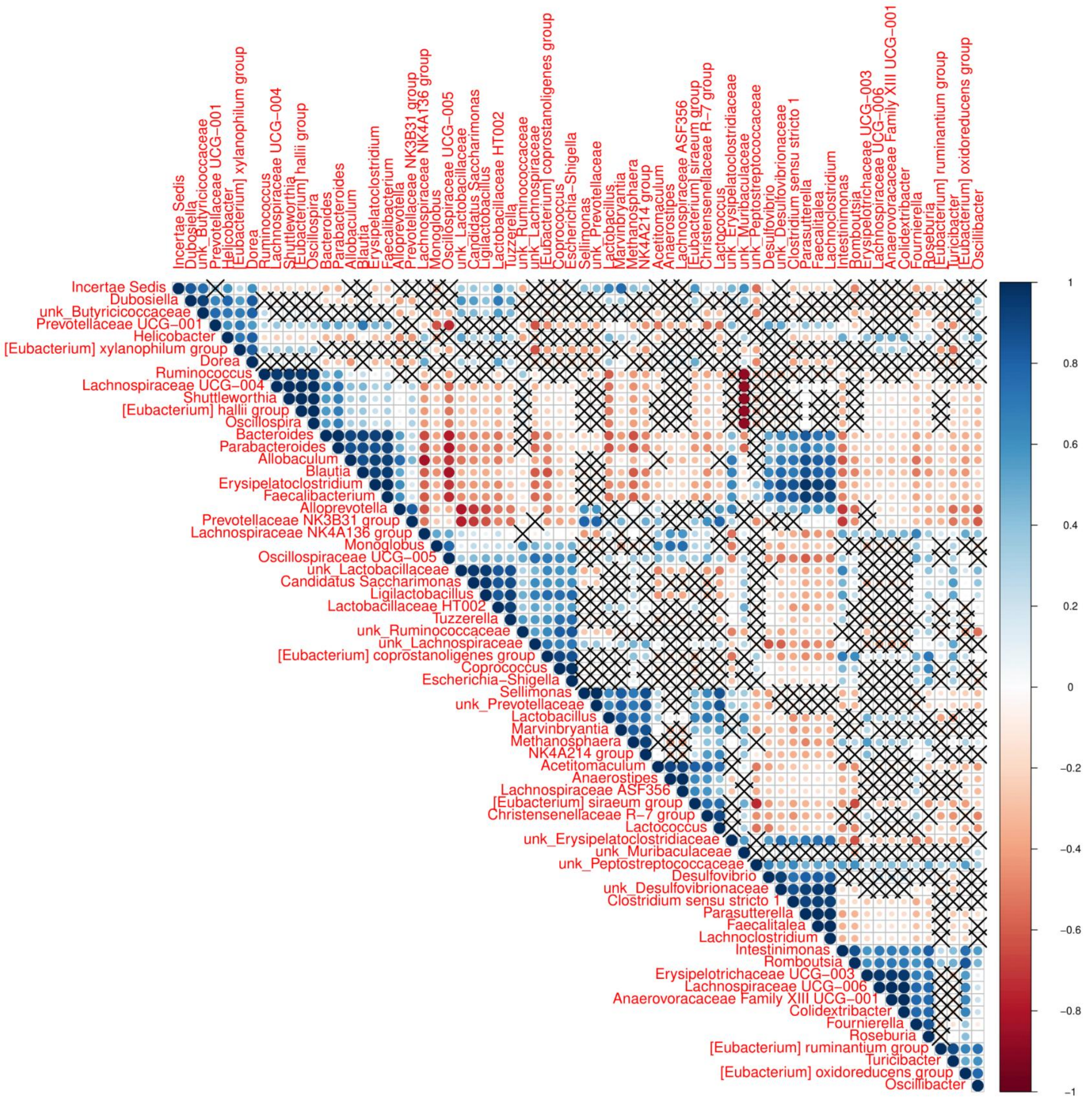

Supplementary Figure S2

Pearson’s correlation by genus abundance in the fecal microbiome of TAAR9-KO rats. Significant values (p<0.05) are shown by cyrcles.
